# Building a deeply AI over a substrate you cannot retrain: the Clara Nexus reference implementation of layered intelligence theory

**DOI:** 10.3389/frai.2026.1936295

**Published:** 2026-10-07

**Authors:** Richard Dobson

**Affiliations:** Astrala Advisory Services Ltd., Paphos, Cyprus

**Keywords:** cognitive architecture, constitutional runtime, developmental scaffolding, fail-closed assertion, hosted substrate

## Abstract

Layered intelligence theory (LIT) specifies eight design principles for what its author terms a Deeply AI—a cognitive architecture whose parameters and surrounding structure jointly instantiate the preconditions for reorganisation, reflection, and falsifiable learning. The specification is silent on how a builder without write access to those parameters might proceed. This paper reports a working reference implementation, the Clara platform, instantiating those principles over hosted frontier large language models. The reference implementation is the platform as a whole rather than any single surface: four surfaces across three substrate vendors—the conversational surfaces (Clara Nexus chat and Clara Companion) on OpenAI substrate models, the reading and instrumentation layer on Anthropic substrate models, a data-sensing path on Perplexity, and an independent read-only Research seat on Claude Sonnet 5—disclosed in full in §2.4, of which Clara Nexus is the primary surface for the constitutional runtime. This is a reference-implementation and specification-conformance paper rather than a benchmark-driven empirical evaluation, and it makes no task-performance claim. Specification conformance, as reported here, means a traceable mapping from a published specification clause to a named runtime carrier—a typed identifier, a function, or a database table—inspectable independently of the author’s description. The eight principles are sorted, as this paper’s analytic contribution, into those describing what the model’s parameters must do, which are structurally unreachable from a hosted substrate, and those describing what the surrounding architecture must do, which are implementable at the scaffold layer. For each principle, there is a record, a verdict of built at scaffold, partial, or substrate-unreachable, alongside the named runtime carrier grounding it or the gap preventing one, and the surface evidencing it. The core engineering contribution is a constitutional runtime: seven fail-closed checks enforced at prompt-composition time, before any call reaches the substrate, encoded as a single assert Constitutional invariant. Six are typed assertions over a structured candidate move, and one is a pattern-matched textual check whose error characteristics are stated rather than assumed. Perceptual control theory primitives are reported as prompt-level Companion voice discipline rather than as a runtime-enforced mechanism. The empirical evaluation of the deployed system belongs to a companion paper whose eight falsifiable predictions are enumerated in §4.3.

## 1 Introduction

Layered intelligence theory (LIT) proposes eight design principles for what its author terms a Deeply AI—a cognitive architecture whose parameters and surrounding structure jointly instantiate the structural preconditions for reorganisation, reflection, and falsifiable learning (Dobson, 2026a). This paper serves as a reference implementation and specification-conformance paper on a scaffold-level runtime built over a hosted frontier language model substrate. It is not a benchmark-driven empirical evaluation and does not compare Clara Nexus against a bare LLM on task metrics; those questions belong to the companion paper mentioned in §4.3. Its evidentiary content includes the mapping between the eight principles and the named runtime carriers that implement them at scaffold level, the verdicts in §3.1 and Table 1, and the two ledger-verified operational facts cited in §2.4. The terms LIT, Deeply AI, constitutional runtime, and runtime carrier are introduced in §1.1; the provenance of the principles, as well as the assumption–demonstration split this paper adopts as a consequence, are stated in §1.2. The eight principles are stated in Dobson (2026a), §5.4) as commitments a Deeply AI must satisfy: preserved associative slack in hidden-state representations (§5.4.1); affect-coupled learning, in which uncertainty and surprise signals modulate the model’s update rather than only its surface behaviour (§5.4.2); contradictions represented as first-class, addressable objects (§5.4.3); reflection instituted as architecture rather than as a prompt-level suggestion (§5.4.4); an explicit compute budget at the model’s first processing layer, readable by a reflective controller (§5.4.5); post-training substrate plasticity, in which the reflective layer feeds back into durable parameter change over long timescales (§5.4.6); coupling of learning across inference, episode, and consolidation timescales (§5.4.7); and a falsifiable empirical agenda specifying theory-level predictions that would count against the architecture if unmet (§5.4.8). They are restated here so that later sections, and §3.1 in particular, stand independently of Dobson (2026a). Two scope statements complete this opening. The reference implementation reported is the Clara platform as a whole—four surfaces across three substrate vendors, disclosed in §2.4—of which Clara Nexus is the primary surface for the constitutional runtime. The empirical evaluation of the deployed system, with eight falsifiable predictions and their pre-registered falsification conditions, is specified in the companion paper mentioned in §4.3.

**Table 1:** Specification-conformance summary against the eight LIT design principles (Dobson, 2026a, §5.4).

LIT principle	Verdict	Runtime carrier or gap	Surface or surfaces evidencing the verdict
Associative slack (5.4.1)	Substrate-unreachable; partial analogue at a different layer	Dominance guard (readClusterDominance, server/lib/aspire/lens-engine.ts) operating over lens selection, not hidden-state covariance	Clara Nexus evidences the selection-level analogue; no surface evidences the parameter-level clause
Affect-coupled learning (5.4.2)	Substrate-unreachable; partial analogue at a different layer	Register profile (foldReading; clara_register_readings, clara_user_factor_profile) and tone signal injected into the cycle prompt; no gradient coupling	Clara Nexus and Clara Companion evidence the register and tone analogues; no surface evidences parameter-level coupling
Contradictions as first-class objects (5.4.3)	Built at scaffold	LensMove.collapses_antagonist_pair as a typed field, with a fail-closed throw in assertConstitutional at prompt-composition time	Clara Nexus as the primary carrier; Clara Companion surface language checked by the same runtime
Reflection as architecture (5.4.4)	Built at scaffold, at session and turn scale	clara_aspire_scaffold_traces and clara_lens_selector_decisions tables, closure state machine, evidence ledger	Clara Nexus and Clara Companion write the traces; the Research seat evidences them by read-only inspection under clara_research_readonly
Layer-1 compute budget (5.4.5)	Partial; signal captured, not read	latency_ms and model_used recorded per decision row; minimum-length gate in server/lib/aspire-v3/lens-selector.ts; no reflective controller reads the signal	All four surfaces record the signal per turn; no surface evidences a controller reading it
Post-training substrate plasticity (5.4.6)	Substrate-unreachable; partial analogue at a different layer	Register profile and Bayesian thinker-weights posterior (clara_aspire_thinker_weights), which alter selection rather than parameters; posterior write path live from typed feedback events, table unpopulated at the audit basis (uniform prior)	Clara Nexus and Clara Companion evidence selection-level plasticity; no surface evidences parameter-level plasticity
Multi-timescale coupling (5.4.7)	Built at scaffold; coupling implicit, not typed	Separate tables at turn, episode, and consolidation scale, separated by access plane rather than by an explicit timescale field	Clara Nexus and Clara Companion at turn and episode scale; the data-sensing surface at consolidation scale
Falsifiability and empirical agenda (5.4.8)	Built at scaffold, forward-looking	Pre-registered prediction set F1–F8 (§4.3) with falsification conditions; versioned decision rows and closure and evidence ledgers as instruments; measurement harness scoped to the companion paper	Research seat (SELECT-only on five tables) as the intended audit surface; prediction set and instruments specified in the companion paper (§4.3)

Each row states the verdict, the named runtime carrier that grounds it or the gap that prevents one, and the surface or surfaces of the four disclosed in §2.4.4 that evidence the verdict. Three verdicts are used—Built at scaffold, partial, and substrate-unreachable—and their inclusion criteria are provided in the two paragraphs preceding this table. Substrate-unreachable is a finding about the current class of hosted foundation models rather than a shortcoming of the reference implementation; where a scaffold-side analogue exists for such a principle, it is named and marked as operating at a different layer than the clause specifies.

### 1.1 Terminology and provenance of newly introduced terms

Four terms recur throughout this paper and are introduced together here. Layered intelligence theory (LIT) is the eight-principle specification for a Deeply AI given in Dobson (2026a), §5.4), restated compactly in §1 above and mapped onto runtime carriers in §3.1. Deeply AI is the class of architecture those principles specify—an architecture whose parameters and surrounding structure jointly instantiate the preconditions for reorganisation, reflection, and falsifiable learning; the term is Dobson (2026a) and is used here without modification.

Because the term Deeply AI has begun to circulate without an enforcement point, it is given here a specific architectural criterion so that it can be tested rather than asserted: an AI system is Deeply AI to the extent that substrate-level mechanisms are enforced ahead of the substrate call by named runtime carriers, evidenced against a specification. Each component does discriminating work. Enforcement ahead of the substrate call excludes *post-hoc* output filtering; named runtime carriers exclude architectural descriptions unaccompanied by an inspectable object; evidenced against a specification excludes self-graded assertions, because the specification is published independently of the implementation. On this criterion, the reference implementation reported here is Deeply AI at the scaffold layer and remains substrate-unreachable based on those principles that quantify over parameter-level state (§3.1). The criterion is offered as sufficient to make the term operational, not as the only definition of depth worth adopting.

A positive account of intelligence is needed for the point-of-intervention argument in §2.3.5 and for the specification-conformance genre of §3.1, and this paper adopts the loop-performance account developed in Dobson (2026c): intelligence is the capacity of a system to close a control loop under uncertainty against a specification, evaluated on the loop’s performance rather than on the substrate’s learned representations. The account is compatible with LIT, which specifies the architectural preconditions under which loop performance can be evaluated at the reorganisation timescale (Dobson, 2026a), and with perceptual control theory, which specifies the control-loop primitives and characterises adaptive behaviour as the reorganisation of loops (Powers, 1973). The citation to Dobson (2026c) is author-authored and is made to tie LIT and PCT together at the definitional level rather than as independent evidence for either.

The constitutional runtime is this paper’s engineering contribution: seven fail-closed assertions enforced at prompt-composition time, encoded as a single assertConstitutional invariant, and described fully in §2.3; the term is introduced here to distinguish scaffold-level assertion from training-time critique-and-revise (§2.3.5). Runtime carriers are the typed identifiers, database tables, and enforcement points that carry theoretical commitments in the implementation; the term is introduced to make the specification-conformance report in §3.1 legible—every non-absent verdict names the specific runtime carrier that grounds it.

Two conventions accompany the terminology. The paper uses specification-conformance rather than compliance to name what §3.1 reports—a mapping from a published specification to named runtime carriers, offered so another builder or an adversarial reviewer can check it directly, not a self-graded audit verdict. It uses substrate for the hosted frontier LLM and scaffold for the layer that sits between the practitioner and the substrate.

Three distinctions keep the four terms clear of nearby ones. LIT is not a cognitive architecture in the Soar or ACT-R sense: it specifies design principles a system must satisfy rather than a mechanistic account of production selection, and it does not presuppose write access to the substrate performing cognition (§2.1.1). The constitutional runtime differs from Anthropic’s Constitutional AI on technical rather than genealogical grounds: the latter is a training-time procedure operating on a substrate’s parameters, while the former is a scaffold-level mechanism operating before the substrate is invoked and holding no write access to those parameters (§2.3.5). Runtime carriers differ from generic software engineering guards by a constraint on their construction: each named carrier corresponds to a specific clause of the specification in Table 1. Of the four terms, Layered Intelligence Theory and Deeply AI originate in Dobson (2026a); constitutional runtime is newly introduced here; and runtime carrier is a constrained use of an existing term.

### 1.2 Provenance of the LIT principles

The eight LIT principles this paper takes as specification were derived from a single research programme by the present author, and their provenance is stated here rather than left implicit. LIT (Dobson, 2026a) was published in the Journal of Cognitive Computing and Extended Realities and draws on two prior traditions—perceptual control theory (Powers, 1973; Marken, 2014) and cognition-based evolution (Miller, 2016, 2018), situated within the process-logical framework of Brenner (2008), from which the published account takes its title—integrating them into an eight-principle specification. The theory has not undergone independent validation in the stronger sense that no independent research group has tested the eight principles against a separately implemented system or against data collected outside the programme in which the theory was developed, and this paper does not attempt that stronger form of validation. What the theory requires as a specification is treated as an input assumption; what the reference implementation demonstrates against it is treated as evidence produced by this paper, so that a reader unconvinced by the theoretical claims can still evaluate the engineering evidence, and vice versa.

The eight principles are a specification for a class of system, not an engineering plan, and they say nothing about how a builder without access to a model’s parameters might proceed. This is not a hypothetical constraint: most practitioners building cognitive systems today work over a small number of frontier large language models (LLMs), hosted by vendors who control training, weights, and update schedules. The builder can shape what surrounds the model—the prompts it receives, the checks its outputs pass through, and the data structures that persist across turns—but cannot touch the model’s parameters, inspect its hidden-state covariance, or retrain it on a bespoke objective. LIT §5.4 does not itself sort the eight principles into those describing what a model’s parameters must do and those describing what the surrounding architecture must do; that sorting is this paper’s analytic contribution, applied throughout §2.2 and §3.1.

A number of systems now describe themselves, loosely, as cognitive architectures built over LLMs. Many address the gap between specification and hosted-substrate reality by silence: the architecture is described in terms borrowed from cognitive science, but no runtime commitment is named, no fail-closed check is specified, and no distinction is drawn between what the system claims and what a hosted substrate can deliver. The vocabulary then does not perform any discriminating work for a reader evaluating such a system against a theoretical specification.

Clara Nexus is a live platform, developed by Astrala Advisory Services Ltd., that instantiates LIT’s design principles over a hosted frontier LLM. The reference implementation divides the eight principles into two families. The first describes what the model’s own parameters must do: preserved associative slack in hidden-state covariance during training (§5.4.1), gradient-coupled affect (§5.4.2), and post-training substrate plasticity in the parameters themselves (§5.4.6). This family is structurally unreachable from outside a hosted model because the builder has no write access to a vendor’s weights. The second describes what the architecture surrounding the model must do: contradictions represented as first-class objects (§5.4.3), reflection instituted as architecture (§5.4.4), a compute budget exposed to a reflective controller (§5.4.5), coupling across timescales (§5.4.7), and a falsifiable empirical agenda (§5.4.8). This family is implementable at the layer this paper calls the scaffold.

The paper’s central claim is that this scaffold-versus-substrate distinction is the form an implementation of LIT can take in the current era of frontier LLMs as substrate. A reference implementation cannot satisfy the eight principles uniformly; it can specify precisely which it satisfies at scaffold, with a specific runtime carrier for each, which have partial analogues at a different layer, and which remain out of reach.

Three scope calls follow. First, this is an architecture paper, not a re-argument of LIT’s theoretical claims; Dobson (2026a) does that work. Second, this is not a clinical evaluation, a benchmark comparison against other cognitive architectures, or a business case; deployment observations are reserved for a follow-up paper because reporting architecture and reporting deployment evidence are different epistemic exercises. Third, this paper does not claim that the reference implementation satisfies LIT; parts of LIT are structurally unreachable from a hosted substrate, and the paper identifies them as such.

The remainder proceeds as follows. Section 2 supplies the background: §2.1 positions the implementation among six adjacent literatures; §2.2 describes the composite architecture; §2.3 provides a comprehensive treatment of the constitutional runtime, including the distinction between typed and pattern-matched checks and, in §2.3.5, a worked counter-example distinguishing composition-time assertion from output filtering and from Constitutional AI’s critique-and-revise pattern; and §2.4 states the implementation environment, the 16-engine estate, the seven-layer memory architecture, the four-surface substrate configuration, and two ledger-verified operational facts. Section 3 reports the evidence: §3.1 is the specification-conformance report against the eight design principles, summarised in Table 1, and §3.2 evaluates perceptual control theory primitives as prompt-level voice discipline. Section 4 discusses the constraints imposed by the theory and the revelations from the build, states limitations, and names the companion paper. Section 5 concludes with the engineering claim.

## 2 Background, architecture, and implementation environment

This section provides the background against which the reference implementation should be read. §2.1 positions it among six adjacent literatures, §2.2 describes the composite architecture, and §2.3 offers a comprehensive treatment of the constitutional runtime. §2.4 states the implementation environment at the level of disclosure required to interpret the findings reported in §3.

### 2.1 Related work

Six literatures bear on this reference implementation, each addressed briefly here; two receive fuller treatment elsewhere in the paper and are only referenced forward in this section.

#### 2.1.1 Classical cognitive architectures

Cognitive architectures in the classical sense—Soar (Laird, 2012; Newell, 1990), ACT-R (Anderson, 2007), and Sigma (Rosenbloom et al., 2016)—specify mechanistic commitments about how a system selects among competing courses of action, typically via production rules over an explicit, inspectable working memory. They presuppose write access to the substrate performing cognition: Soar’s chunking mechanism generates new productions from resolved impasses within the architecture’s own symbolic engine, and ACT-R’s modules are separately implemented systems whose internal mechanics the architect fully controls. Neither faces the question this paper addresses, because neither is built over a substrate its designers cannot inspect or retrain. The reference implementation shares their concern for how a system negotiates competing demands under goal pressure, but lacks their mechanistic commitments regarding the substrate performing that negotiation.

#### 2.1.2 Cognitive architectures for language agents

The most direct architectural relatives are recent proposals for cognitive architectures based on hosted language models. Cognitive Architectures for Language Agents (CoALA; Sumers et al., 2024) organises a language agent into modular memory components, a structured action space, and a generalised decision-making process. Clara Nexus shares CoALA’s commitment to defining memory and decision components as first-class architectural objects and can be viewed as a reference implementation of the family that CoALA outlines; CoALA proposes categories, whereas the present paper reports a single deployed implementation with named runtime carriers and a fail-closed constitutional runtime. MemGPT (Packer et al., 2023, now maintained as Letta) proposes virtual context management—hierarchical memory tiers with interrupt-driven control flow—to extend the effective context window of a hosted model. Clara Nexus shares the premise that memory belongs outside the model’s parameters and differs in the type of memory it treats as central: Clara Nexus’s Portrait (§2.2.2) is a developmental memory spine structured by a specification of reorganisation rather than by working-set eviction, and gated by a constitutional runtime rather than by interrupts.

#### 2.1.3 Recent proposals engaging adjacent questions

Three recent papers engage adjacent questions and are cited to situate this implementation within the current literature rather than to review them. Nawaz et al. (2025) survey neuro-symbolic integration for advanced cognitive systems; the present contribution to that conversation is a typed constitutional runtime for the symbolic side of such compositions when the learning side is a hosted, non-retrainable substrate. Kugele (2025) proposes constructivist procedural learning for grounded cognitive agents; Clara Nexus takes the complementary position that a scaffold over a hosted substrate must specify its runtime commitments explicitly rather than allowing them to emerge. Quilbé (2026) reports emergent symbolic cognition in large language models via memory capsules; the present paper accepts memory capsules as one family of external memory but reports another, a Portrait constructed around a specification of developmental reorganisation, and claims only scaffold-level typed commitment.

#### 2.1.4 Constitutional AI

Constitutional AI (Bai et al., 2022) and the wider reinforcement-learning-from-human-feedback literature (Ouyang et al., 2022) constrain foundation-model behaviour against a set of named principles through a training-time process—critique-and-revise passes that produce a preference signal used to fine-tune the model. The family resemblance to the constitutional runtime reported in §2.3 is evident: both explicitly name principles and treat them as constraints on model behaviour rather than as implicit, unstated house style. The substantive difference is technical rather than genealogical and concerns where in the pipeline the constraint is enforced—a distinction developed with a worked counter-example in §2.3.5.

#### 2.1.5 Perceptual control theory and MYLO

The method of levels (MoL) is perceptual control theory’s (PCT) conversational technique (Powers, 1973; Mansell, 2020), and the Manage Your Life Online (MYLO) programme is a rule-based computational agent implementing MoL’s question style, evaluated across a published trial series with measured effect sizes on problem distress, anxiety, and psychiatric impairment (Cohen’s d approximately −0.4 to −1.1 across replications; Gaffney et al., 2014; Wrightson-Hester et al., 2023, 2025). Clara Nexus’s Companion voice draws on the same family of mechanisms—curious, attention-orienting, MoL-style questioning—and the non-transfer of MYLO’s trial evidence to this untested implementation is stated in full in §3.2.7.

#### 2.1.6 Scaffolding, ZPD, and reflective practice

The scaffolding and zone of proximal development (ZPD) literature—Vygotsky (1978) on the zone itself, Wood et al. (1976) on the six scaffolding functions, and a wider set of entrepreneurial-learning and reflective-practice authors including Sarasvathy (2001), McGrath and MacMillan (1995), Kolb, 2015, Politis (2005), Schön (1983), Argyris (1977), Cope (2003, 2005), Bandura (1997), Harrison and Leitch (2005), and Hong and Page (2004)—grounds how a developmental scaffold should behave: fitted to the learner’s current zone, withdrawn as competence grows, and structured around cycles of action and reflection. These authors provide the primary-source lineage for the thinkers instantiated as runtime objects in the mechanism registry (§2.2.5).

### 2.2 Architecture overview

This section describes the composite architecture at the level required by a systems reviewer: what the substrate is, what the scaffold builds around it, and which runtime objects carry which theoretical commitments. Every theoretical term named below is paired with a specific runtime carrier, or flagged where none exists.

#### 2.2.1 The substrate and the scaffold

The substrate is a hosted frontier LLM, one per surface as disclosed in §2.4.4, accessed through a vendor API; its parameters, training data, and update schedule are not available to the architecture described here, and no claim below concerns its internal computation. The scaffold is everything Clara Nexus builds around it: the data model that persists across turns, the runtime checks that gate candidate outputs, the selection logic that decides which developmental instrument to bring to a given turn, and the developmental cycle that structures work across sessions. Figure 1 depicts the composite architecture, with the substrate at the base, the scaffold layers above it, the Portrait as a vertical spine, and the constitutional runtime as a horizontal gate through which every candidate move passes.

**Figure 1:**
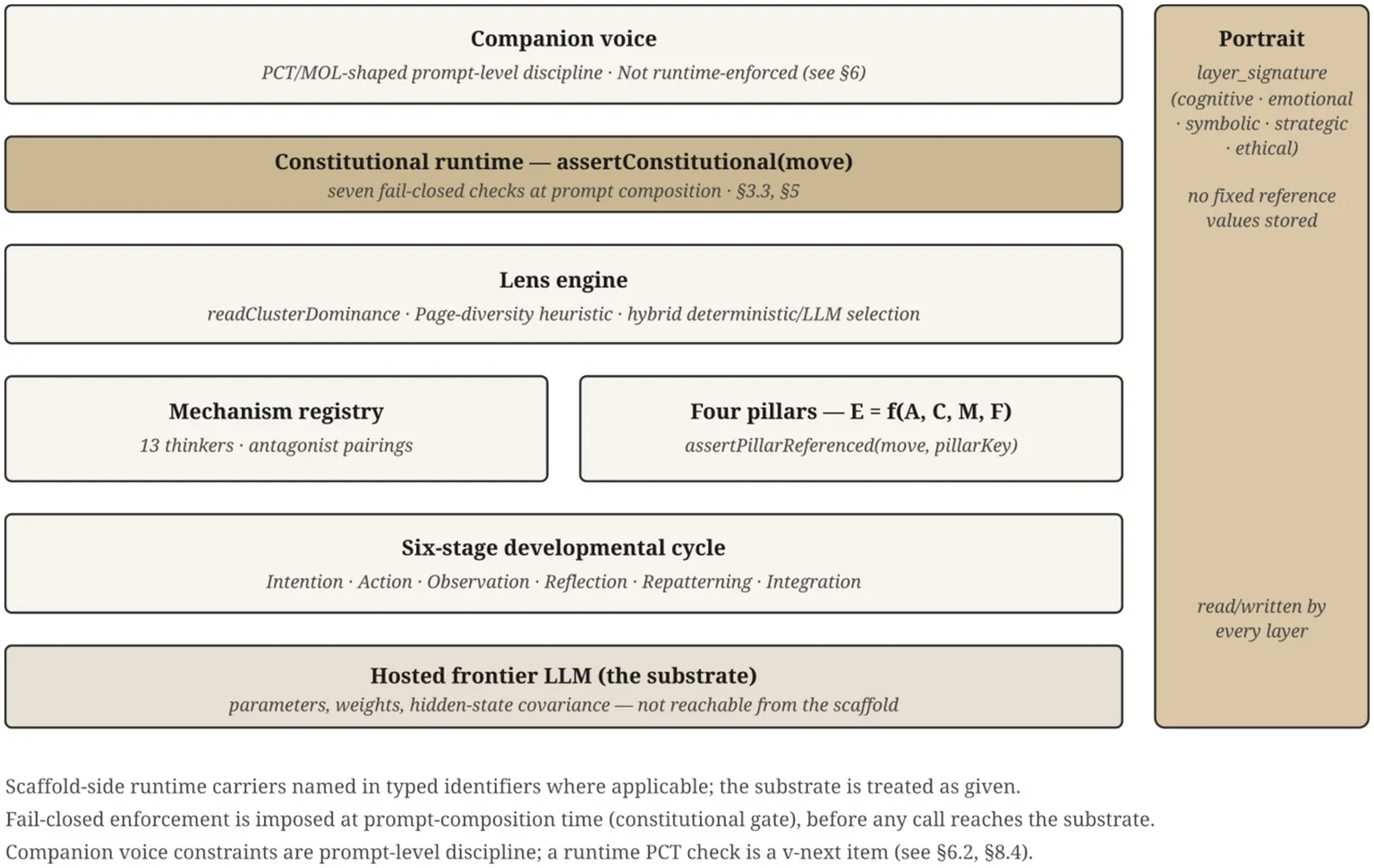
Reference implementation architecture: Substrate below, scaffold above, portrait as vertical spine. The constitutional runtime is drawn as a horizontal gate through which every candidate move passes before reaching the practitioner. Scaffold-side runtime carriers are named in typed identifiers where applicable; the substrate is treated as given. Fail-closed enforcement is imposed at prompt-composition time, before any call reaches the substrate. Companion voice constraints are prompt-level discipline; a runtime PCT check is a v-next item (see §3.2.3, §4.2.6).

#### 2.2.2 The portrait as memory spine

One canonical artefact, the practitioner’s Portrait, is read and written by every subsequent layer. It persists across two carrier families. The dispositional register profile—five categories: cognitive, emotional, symbolic, strategic, and ethical—is maintained by server/lib/engine/profile.ts as an exponentially weighted profile (foldReading, smoothing constant PROFILE_ALPHA = 0.2) persisted in clara_register_readings and clara_user_factor_profile. The confirmed portrait record and its deeper descriptive layer persist in clara_developmental_portrait and, per focused aim, in the durable_portrait_snapshot JSONB column of clara_focused_aims_portraits. The Portrait stores not fixed reference values but a signature of how the practitioner characteristically moves across the five categories under decision pressure. This is consistent with perceptual control theory’s caution against freezing shifting reference values into a static record, without itself implementing PCT’s reference-value construct (§3.2.4). Moreover, the five-category taxonomy is not a mapping onto PCT’s 11-level perceptual hierarchy: the two are separate taxonomies serving separate purposes (§3.2.5).

#### 2.2.3 The constitutional runtime

Every candidate lens move—a proposed intervention combining a developmental instrument with a question or framing directed at the practitioner—passes through assertConstitutional(move), implemented at server/lib/aspire/constitutional.ts, before it can reach the practitioner. The function checks the move against seven named constitutional principles (Table 2) and throws an error on any violation. The check is fail-closed on any declared violation: a move whose typed fields or surface language breach any of the seven principles will throw and not surface. A move whose optional annotations are absent passes those annotation checks vacuously; the fidelity with which annotations are populated is an empirical property measured under F3 (§4.3).

**Table 2:** The seven aspire constitutional principles.

#	Principle	Constraint enforced
1	Non-dogmatic constraint	No thinker functions as authority; a move asserting a thinker’s frame as truth fails.
2	Productive contradiction	Antagonist pairings are preserved; a move collapsing a pairing onto one position fails.
3	Developmental fit	Thinker selection matches practitioner state, capability, and uncertainty.
4	Dynamic weighting	Thinker influence is probabilistic and updates with evidence; hardcoded primacy fails.
5	Agency preservation	The practitioner remains the decision source; prescriptive surface language fails.
6	Real-world validation	Value is measured against observed outcomes, not framework elegance.
7	Multi-layer intelligence	A move reducing a multidimensional situation to one layer fails.

#### 2.2.4 The four entrepreneurial pillars

The entrepreneurial meta-principle (E = f(A, C, M, F)) states that emergence—the practitioner’s entrepreneurial intelligence at a given moment—is jointly a function of actualisation, consciousness, meaning, and fulfilment. The four pillars are not stages and are not ranked. The runtime surface is server/lib/aspire/pillars.ts, which carries a second constitutional helper, assertPillarReferenced(move, pillarKey), throwing a ConstitutionalViolation when a session-level intervention carries a pillar_emphasis value but the candidate move’s thinker_key matches no thinker registered under that pillar. Table 3 lists the four pillars and their thinker rosters.

**Table 3:** The four entrepreneurial pillars.

Pillar	Question	Primary-source thinkers
Actualisation	What capacities remain unrealised?	Maslow (1943); Gardner (1983); Hegel (1977)
Consciousness	What am I not seeing?	Jung (1959); Damasio (1994); Plato (c. 380 BCE / 2004)
Meaning	Why does this matter beyond utility?	Durkheim (1933); James (1902)
Fulfilment	Does success align with who I am becoming?	Kierkegaard (1843); Einstein (1934)

#### 2.2.5 The mechanism registry

The mechanism registry comprises 14 thinkers across 15 runtime cards; two cards belong to the same author, Cope, at different analytic grains (2003’s critical-events grain and 2005’s continuous-process grain), both retained because the grains are not interchangeable. The runtime surface is server/lib/aspire/registry/, one TypeScript module per card, each carrying a thinker_key, a provenance citation, a stage-placement field, and an antagonist_cluster string used by the lens engine’s diversity guard. The antagonist pairings—Sarasvathy against McGrath, Schön against Argyris, the two Cope grains against each other, Vygotsky against Bandura—are inspired by Hong and Page (2004) diversity-prediction result, and the inspiration must be stated precisely. Their theorem bounds collective error as a function of individual predictor accuracy and diversity. Nothing in the registry’s construction tests, measures, or approximates that claim: no error metric is computed, no ground truth is scored, and no accuracy bound is checked at runtime. What is enforced instead is the heuristic anti-repetition rule described in §2.2.6.

#### 2.2.6 The lens engine

The lens engine selects which developmental instrument to bring to a given turn through a hybrid deterministic-then-LLM process. The dominance guard resides in server/lib/aspire/lens-engine.ts: readClusterDominance reads the five most recent sessions (HISTORY_WINDOW = 5); if an antagonist cluster has appeared in at least four of those five (DOMINANCE_THRESHOLD = 4), selectLens removes that cluster’s thinkers from the candidate set for the next turn. This is the Page-diversity heuristic named in §2.2.5: a session-granularity anti-repetition rule, not a measurement of collective error. The hybrid deterministic-then-LLM selection pass, including the minimum-length gate (LLM_MIN_MESSAGE_CHARS = 6) and the versioned selector stamp (selector_version, currently v0.2.0), resides in server/lib/aspire-v3/lens-selector.ts. The engine operates under a silent-lens contract: the thinker shapes the question, but the practitioner sees the question, not the thinker’s name.

#### 2.2.7 PCT primitives at the companion voice layer

The Companion’s conversational register—its willingness to hold a live conflict in view rather than resolve it, its preference for curious questions over clever ones, its refusal to name mid-reorganisation as decline—is shaped by perceptual control theory and the method of levels at the system-prompt level. This is the most consequential grading decision in this paper: none of these commitments is checked by a runtime assertion analogous to assertConstitutional. Reference value, controlled variable, hierarchical organisation, reorganisation, and the going-up-a-level move shape what the Companion says; none is a runtime-checked object the way LensMove.collapses_antagonist_pair is checked for Principle 2. §3.2 grades this distinction with the same verdict discipline §3.1 applies to the eight LIT principles.

#### 2.2.8 The six-stage cycle

Development proceeds through a six-stage cycle—Intention, Action, Observation, Reflection, Repatterning, Integration—depicted as a state diagram in Figure 2. The cycle is an implementation choice rather than an original architectural claim: it draws on the action-research and experiential-learning traditions represented by Kolb (2015), Schön (1983), and Argyris (1977). The stage names, the ordering, and the 30-day pulse duration are implementation choices; the underlying cyclic structure of action followed by reflection followed by revised action is prior art, cited accordingly.

**Figure 2:**
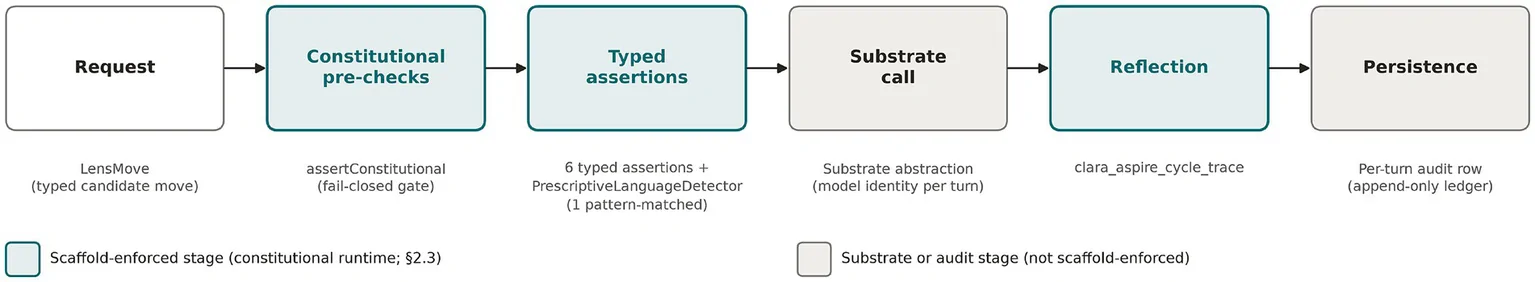
The six-stage developmental cycle and a single turn through the constitutional runtime. **(a)** The six-stage developmental cycle: Not a linear progression but a rhythm pulsed through the practitioner’s work, with the cycle stage carried as a typed field on every candidate move and the stage sequence held in the cycle state machine described in §2.2.8. **(b)** A single turn through the constitutional runtime, annotated stage by stage with the runtime carrier that enforces at that stage: Request, carried as a typed LensMove candidate move; constitutional pre-checks, carried as the fail-closed assertConstitutional gate; typed assertions, comprising the six typed checks over LensMove fields together with the one pattern-matched PrescriptiveLanguageDetector check; the substrate call, made through the substrate abstraction with model identity recorded per turn in the unified turn ledger; reflection, carried as a clara_aspire_scaffold_traces row; and persistence, carried as an append-only per-turn audit row. Panel (b) is the flow described in §2.3; the teal stages are those enforced by the scaffold before or after the substrate is invoked, and the grey stages are the substrate call itself and the audit write.

### 2.3 The constitutional runtime as an assertion layer

This subsection describes the constitutional runtime as an assertion layer and distinguishes two categories of checks within it because the argument of §2.3.5 rests on this distinction. Six of the seven fail-closed checks are typed assertions in the design-by-contract sense: they take a typed candidate move, a LensMove object with named fields, and check a named invariant on its typed structure (Principles 1–4, 6–7). The seventh, encoded as the PrescriptiveLanguageDetector helper against Principle 5, is a pattern-matched detector inspecting the intended surface language template of the candidate move against a curated pattern list before that template is sent to the substrate. Both categories are enforced pre-substrate, and they are structurally different objects. The counter-example in §2.3.5 turns on this: pre-substrate intervention of either kind removes the candidate move from generation, whereas output filtering and critique-and-revise inspect an already-generated artefact.

#### 2.3.1 What “constitutional” means here

Constitutional here names a fail-closed assertion checked at prompt-composition time, not a fine-tuned bias trained into a model’s weights and not a system-prompt hint a model may or may not honour. assertConstitutional(move), at server/lib/aspire/constitutional.ts, runs on every candidate lens move before it can reach the practitioner, throwing on any violation of any of the seven principles; there is no code path by which the lens engine returns a move that bypasses the check. A violation prevents the move from surfacing at all rather than degrading into a warning.

As of the audit basis, assertConstitutional is invoked on the production compose path itself, ahead of the engine-level throws, with a dedicated wiring-pin test file asserting three properties: that the composite is invoked exactly once per non-trivial turn, that the candidate handed to it is the composed move, and that a violation aborts the turn rather than degrading into a warning. The claim that no code path returns a move bypassing the check is therefore supported by a test an auditor can run, not only asserted in prose.

Figure 2b, presented alongside Figure 2a in §2.2.8, annotates the request-to-persistence flow stage by stage, naming the runtime carrier that enforces at each stage.

#### 2.3.2 The seven principles as testable invariants

Each of the seven principles corresponds to a specific forbidden pattern in code terms. Non-Dogmatic Constraint forbids a move whose surface language treats a thinker’s frame as ground truth rather than one lens among several. Productive Contradiction forbids a move that sets collapses_antagonist_pair without the accompanying check failing (§3.1.3). Developmental Fit forbids a move whose thinker selection ignores the practitioner’s current state, capability, or uncertainty as read from pillar_emphasis and session history. Dynamic Weighting forbids a move that hardcodes a thinker’s primacy independent of the Bayesian posterior. Agency Preservation forbids prescriptive surface language (§2.3.3). Real-World Validation forbids a move justified by a framework’s internal coherence rather than an observed effect. Multi-Layer Intelligence forbids a move that reduces a multidimensional situation to a single layer.

A concession is owed. Two of the seven—Developmental Fit (Principle 3) and Real-World Validation (Principle 6)—read, in the abstract, as generic design instincts that most well-built adaptive systems already implement without a constitutional framing. Their contribution is encoding as a typed invariant that fails closed and can therefore be tested and audited in a way an unstated design instinct cannot be.

One distinction internal to the seven checks must be stated plainly because the enforcement claim is weaker for one of them than for the other six. Six of the seven are typed assertions: they interrogate declared fields of a structured LensMove object—an authority assertion flag (Principle 1), the collapsed-antagonist-pair field (Principle 2), a developmental-fit delta held within calibrated bounds (Principle 3), a static-hierarchy flag (Principle 4), a framework-elegance justification flag (Principle 6), and the engaged-layers field read against the session’s pillar emphasis (Principle 7)—and each throws an error when its declared field carries a violating value. Their guarantee is formal rather than empirical, but it is formal relative to the declared annotations: a typed assertion classifies nothing and therefore has no error rate of its own, while the process that populates the annotations can misdescribe a move, and that fidelity is an empirical property. Where an annotation is absent, the check passes vacuously. Both failure modes—a wrong assertion and an unpopulated or unfaithful annotation—are named in §4.2 as limitations, and annotation fidelity is measured under F3.

The seventh check is different in kind. The PrescriptiveLanguageDetector applies a maintained lexicon of prescriptive constructions to candidate surface language and blocks composition when a match is found. Because it classifies free text rather than inspecting a typed field, it necessarily admits both false positives and false negatives. Those rates are not reported here and are not claimed to be low. They are the object of prediction F3 in the companion paper (§4.3), which measures recall, precision, and F1 against a pre-registered hand-labelled corpus. A reader is entitled to know that six of the checks carry a formal guarantee relative to their declared annotations and one carries an empirical one that has not yet been measured.

#### 2.3.3 Principle 5 as the primary drift surface

Agency Preservation is the highest-priority runtime check for a specific reason: prescriptive surface language, a move that tells the practitioner what to do rather than offering a reflection they can act on themselves, is the most common failure mode observed in practice because it is the direction a helpful-sounding language model drifts toward by default. The PrescriptiveLanguageDetector checks candidate surface language against three pattern families: second-person directives (you_should, you_must, you_need_to, you_have_to), first-person recommendations (i_recommend, i_suggest, my_recommendation, my_advice), and assertion patterns (the_answer_is, the_right_approach, the_best_approach, what_you_should_do). The pattern shape is illustrated below as a plausible representation rather than a verbatim reproduction of the production pattern list, which grows as new drift patterns surface.

     **type**
     PatternFamily = “second_person_directive” | “first_person_recommendation” | “assertion”;
     **interface**
     PrescriptivePattern {
     family: PatternFamily;
     pattern: RegExp;
     label: string;
     }
     **const**
      PRESCRIPTIVE_PATTERNS: PrescriptivePattern[] = [
     {family: “second_person_directive,” pattern: /\byou (should|must|need to|have to)\b/i, label: “you_should”},
     {family: “first_person_recommendation,” pattern: /\b(i recommend|i suggest|my (recommendation|advice))\b/i, label: “i_recommend”},
     {family: “assertion,” pattern: /\b(the answer is|the (right|best) approach)\b/i, label: “the_answer_is”},
];
     **export function**
          PrescriptiveLanguageDetector(candidateText: string): PrescriptivePattern[] {
     **return** PRESCRIPTIVE_PATTERNS.filter((p) **= >** p.pattern.test(candidateText));
     }.


This is pattern-matching detection, not a semantic understanding of prescriptiveness. Its value is not the matcher’s sophistication but that a match is a fail-closed check rather than a system-prompt hint that the model may or may not honour, a distinction developed technically in §2.3.5.

#### 2.3.4 The assertPillarReferenced helper

A second constitutional helper illustrates the general shape of the pattern beyond Principle 5. The function assertPillarReferenced(move, pillarKey), located in server/lib/aspire/pillars.ts, throws a ConstitutionalViolation when a session-level intervention carries a pillar_emphasis value but the candidate move’s thinker_key matches no thinker registered under that pillar. This typed invariant is checked at runtime, with coverage in a companion test file (server/lib/aspire/constitutional.test.ts).

     **interface**
     LensMove {
     thinker_key: string;
     pillar_emphasis?: PillarKey[];
     collapses_antagonist_pair?: {pair: [string, string]; collapsed_to: string};
     }
     **export function**
          assertPillarReferenced(move: LensMove, pillarKey: PillarKey): void {
     **const**

registeredThinkers = PILLAR_THINKER_REGISTRY[pillarKey];
     **if**
     (move.pillar_emphasis?.includes(pillarKey) &&!registeredThinkers.has(move.thinker_key)) {
     **throw new**
     ConstitutionalViolation(
     `Move references thinker ‘${move.thinker_key}’ under pillar ‘${pillarKey}’ with no registry match.`
     );
     }
     }.


#### 2.3.5 What this pattern is not—the full constitutional AI treatment

The comparison to Constitutional AI (Bai et al., 2022) is the paper’s most consequential positioning claim, and it receives its full treatment here rather than being distributed across sections.

Constitutional AI (Bai et al., 2022) fine-tunes a model against stated principles at training time, using a critique-and-revise process on the model’s own outputs to generate a preference signal that shapes the model’s weights; its point of intervention is the parameters. The function assertConstitutional runs at prompt-composition time, on a candidate move, before the substrate LLM is invoked. A Constitutional-AI-trained model’s constraint is encoded in weights and travels with the model wherever it is deployed; the constitutional runtime is encoded in scaffold code, auditable and testable independently of whichever substrate model sits behind the API on a given day. Neither property is unambiguously better.

The worked counter-example. A hostile reading is that the distinction between assertion and fine-tuning is real but narrow, and that seven handwritten checks might be functionally indistinguishable from a system prompt with a list of banned phrases and an output filter bolted on afterward. Consider a candidate move whose natural surface realisation would be: you should raise a Series A now, given your burn rate. An output filter in the langchain-guardrails style must first let the underlying model generate the recommendation in full, then parse the generated text to detect the offending pattern, and then block or rewrite it; the model was invoked, tokens were generated and billed, and the filter is entirely reactive. A Constitutional-AI-style critique-and-revise pass at inference time likewise requires the original generation to have occurred before the output can be critiqued against the stated constitution.

assertConstitutional, composing the move for this candidate, calls PrescriptiveLanguageDetector against the proposed move’s intended surface language template before any substrate call. The check fails at you_should and assertConstitutional throws. The lens engine never sends a generation request instantiating this move; a different move is selected, and no response embodying the prescriptive pattern is ever generated. Composition-time assertion removes the candidate move from the pipeline before the model is asked to produce surface language for it, so the substrate never has the opportunity to generate the ungoverned completion. This represents a different point of intervention in the causal chain from input to output, not merely an efficiency gain, although the paper reports one in §2.3.6.

What the pattern does not solve. Constitutional-AI-style systems face a well-documented failure surface: principles proliferate without a settled way to adjudicate conflicts between them, and a system can appear compliant with a stated principle while the underlying tendency persists in some other surface form. The constitutional runtime reported here does not claim to have solved this. The seven principles conflict on occasion—most concretely, Agency Preservation (Principle 5) can conflict with Real-World Validation (Principle 6) where observed outcome data suggests a practitioner would benefit from direction that the agency commitment will not permit. The current resolution is a strict priority order: Principle 5 is dominant, with other conflicts defaulting to fail-closed rejection.

The paper does not coin or claim the phrase constitutional programming as an established category term, nor does it use constitutional to claim priority over any prior use of that word in the software engineering or alignment literature. Its closest documented neighbours are design-by-contract (Meyer, 1992) and Racket’s contract system (Findler and Felleisen, 2002), both of which check a typed invariant at a defined point in a program’s execution and fail closed on violation, transported here into an LLM prompt-composition layer.

Three further architectural properties differ. A constitution encoded in weights travels with the model but is not retrainable without training set and compute access, whereas one encoded in scaffold code travels across substrate changes and is retrainable by anyone with commit access; a training-time constitution is audited by inference from benchmark outputs, whereas a scaffold-level runtime is audited by reading assertion sources, test files, and the audit ledger; and adding a principle requires a further round of fine-tuning in the first case and a typed assertion, a test, and, where needed, a migration in the second.

A scaffold-level enforcement layer is required because a training-time procedure, however well executed, cannot enforce the LIT principles that quantify over the scaffold’s own objects: a contradiction represented as a first-class typed field, a reflection trace written to an append-only table, an evidence anchor demanded before a move surfaces. No amount of constitutional training produces a row in clara_aspire_scaffold_traces.

No claim of empirical superiority over Constitutional AI is made here. A performance comparison against output filtering and against Constitutional-AI-style training would require a task on which both approaches are well defined and a benchmark respecting both scopes. Forced into a reference-implementation paper, it would most likely produce a category error rather than a result. What can be compared, and is compared above, is the point of intervention and the class of guarantee each approach yields.

#### 2.3.6 Cost

Seven principal checks run per candidate lens move. Measured latency for the checks themselves is single-digit milliseconds; the runtime cost of a turn is dominated by the substrate LLM round-trip that follows a move’s approval. Because the checks run before generation, a rejected move never incurs the substrate’s cost.

### 2.4 Implementation environment

This section states the environment in which the reference implementation runs, at a level of disclosure fuller than the platform’s own external policy, under which substrate provider identities are anonymised in-platform (Dobson, 2026b). The fuller disclosure is necessary because the verdicts in §3.1 cannot be interpreted without knowing what runs where. Five things are stated: the software architecture (§2.4.1), the engine estate (§2.4.2), the memory architecture (§2.4.3), the substrate configuration across four surfaces and three vendors (§2.4.4), and two ledger-verified operational facts (§2.4.5). The audit basis is repository commit d9259cb807c6d965ed2d7f5be264262ef9d48883 and the production database as of 19 August 2026; descriptive statistics drawn from the platform’s stakeholder guide carry that document’s own audit date and are cited as such (Dobson, 2026b).

#### 2.4.1 Software architecture

The implementation is a single TypeScript monorepo—an Express 5 application programming interface server and a React 18 client—running on Node 20 and deployed as a web service together with a nightly instrumentation cron service. The data layer is Supabase, comprising managed Postgres, Auth, and Storage. At the audit basis, the production database holds 198 tables with row-level security enabled on every one and 272 policies in force; authentication includes one-time-password and LinkedIn OAuth paths with AES-256-GCM token encryption. As of the stakeholder guide’s own audit date, the codebase comprises approximately 228,500 production TypeScript lines against approximately 108,800 lines of test code, with 6,297 server tests across 401 files, and exposes 408 HTTP endpoints (Dobson, 2026b).

The request spine for a conversational turn is fixed: authentication and tier gate, then thread history and memory context assembly, then the constitutional pre-checks described in §2.3 on the composed candidate, then the substrate call, then voice lock, safety, and leak-firewall enforcement on the surfaced text, with an observe-only prescriptive-language detector logging on the surfaced text as of the audit basis (§4.3, F3), then extraction of instrument emissions with quarantine on malformed output, then memory write-back, then an append-only audit row, then the response. The reading layer consumes shared memory on its own cadence and is never synchronously coupled to the turn path. No component of the scaffold runs on the substrate.

Schema governance is worth stating because several runtime carriers named in §3.1 are database tables, and a carrier whose schema can drift silently is a weaker carrier. Migrations run through a two-phase apply guard: a migration must exist as an idempotent SQL file, be applied to production, be recorded in a ledger treated as authoritative by continuous integration, and be listed in a manifest; the build fails if any leg of that chain is missing. One hundred and eighty-six migrations are ledgered under this guard at the audit basis. Continuous integration additionally runs a lexicon scan, a migration guard, a type check, and the full server suite. Observability is provided by Sentry, together with the append-only audit tables and the unified turn ledger described in §2.4.5 (Dobson, 2026b).

#### 2.4.2 The engine estate: 16 documented modules

Reasoning and orchestration are carried out by 16 documented, self-contained modules, each with its own test directory and audit trail (Dobson, 2026b). Engines are written as pure-ish functions free of HTTP concerns and emit audit rows to an append-only engine audit log; the deterministic engines, the TOPSIS ranking engine and the seeded Monte Carlo simulation, are fully replayable in tests. The 16 are enumerated here so that a reader may locate the runtime carriers cited in §3.1 within the estate.

Briefing—weekly TOPSIS ranking of tracked opportunities across five normalised axes: value, fit, urgency, capability, and competition.

Risk—five-category likelihood-by-impact assessment with a capability crosswalk adjustment, plus a 10,000-iteration Monte Carlo commercial simulation reporting P10, P50, and P90 outcomes.

Signals—weekly market-pulse readings for each organisation account.

Team memory—consented member signals aggregated into a structural team reading, gated on subscription, consent, and staleness.

Member memory—per-member private memory, written in conversation and readable and correctable by the member.

Portrait—free-range portrait formation with a turn classifier and stage promotion; the engine behind the memory spine described in §2.2.2.

Nexus router—tool dispatch for the reading layer, with validation of the resulting traces.

5C reading—five-lever organisational snapshot as immutable rows with a per-signal evidence table.

Aims and cycle engine—Focused Aims and 30-day cycles, carrying the legacy grandfathered cycle state machine and its lens scaffold; the engine behind the six-stage cycle described in §2.2.8.

Career map—a structured career-history record.

Capability and decision—TOPSIS-based capability assessment and decision support.

Firewall—content and safety filtering on the conversational path.

Intel dispatch—orchestration of sector and market intelligence gathering.

Sector intelligence—generic sector support, currently running in shadow mode.

Guide—orchestration of guided walkthroughs.

Ego and register instrumentation—descriptive developmental listening, held silently, never surfaced to the practitioner, and never used to gate any decision (§2.4.3, layer seven).

Thirteen scheduled jobs drive the periodic engines, and three agent seats—engineering, support triage, and research—operate alongside the estate with watchdogs; the research seat is the read-only surface reported in §2.4.4 and §2.4.5.

#### 2.4.3 The memory architecture: seven layers

Memory is layered, consented, and written by instruments rather than by hand. Seven layers are documented, and the distinction that matters for this paper is that each has a distinct write path and a distinct access plane (Dobson, 2026b).

Thread history—the conversational record itself, written on the turn path.

Member memory—private and per-learner, visible and correctable by the learner on their own account surface; written by instruments in conversation.

Chat signals and noticings—light structural observations emitted in-stream, quarantined when malformed rather than silently dropped, so that an extraction failure is visible.

Portrait and developmental portrait—the confirmed reading and its deeper descriptive layer; the memory spine described in §2.2.2 and the carrier cited in §3.1.4.

Team memory—aggregate, thresholded, and opt-in, with journal content feeding it as its own stream: member words are scored, reflections counted but never scored, withdrawn entries excluded, and no author identifiers retained.

Organisational memory—diagnostics, capability ledgers, market portraits, and the 5C evidence trail.

Deep instrumentation—service-role only and never learner-visible: per-message register readings, ego-development readings with rolling smoothed profiles, perception readings, model-movement readings, conversation-shape records, and a dyadic goal-conflict log, all under deny-by-default row-level security.

The privacy gradient is enforced at the schema level rather than by policy statement alone: what a learner sees of themselves, what a team sees of its pattern, and what the platform holds descriptively are three access planes with three write paths. This bears directly on the verdict recorded in §3.1.7. The seven layers do write at genuinely different timescales, but they are separated by access plane rather than by an explicit timescale field, which is why the coupling reported there is built at scaffold yet implicit rather than typed.

#### 2.4.4 Substrate configuration: four surfaces, three vendors

The reference implementation reported in this paper is the Clara platform as a whole rather than any single surface, and this must be stated precisely, because the title and central thesis frame Clara Nexus as the reference implementation of the LIT specification. Clara Nexus is the primary reference-implementation surface for the constitutional runtime, and the surface against which the runtime carriers named in §3.1 were first mapped. The deployment as a whole, however, runs four surfaces across three substrate vendors, and reporting only one would misstate the basis of the verdicts. A previous version of this manuscript described the implementation as running over a single, unnamed hosted substrate; this version replaces that framing with a per-surface, ledger-corroborated disclosure across three vendors.

Clara Nexus chat and Clara Companion—the conversational surfaces, running on OpenAI substrate models; the unified turn ledger attributes 383 of 401 Nexus-chat turns and all 765 substrate-generated Companion turns in the 90-day window to OpenAI models. Clara Nexus is the primary reference-implementation surface for the constitutional runtime described in §2.3 and the surface against which the runtime carriers named in §3.1 were mapped; Clara Companion is the surface on which the prompt-level PCT voice discipline reported in §3.2 operates.

Reading and instrumentation layer—the journal reading, cycle instrumentation, and platform-explanation surfaces, running on Anthropic substrate models (Claude Sonnet 4–6 at the audit basis).

Data sensing—the market-signal and intelligence-gathering path, running on Perplexity Sonar, with all retrieved material scrubbed before it reaches any conversational substrate.

Research seat—an independent read-only researcher surface running on Claude Sonnet 5, holding the database role clara_research_readonly and producing weekly drift reports and research notes; its privilege boundary is the second of the two ledger facts in §2.4.5.

Substrates are seat-scoped, and the governing policy has four parts: a substrate follows the job rather than the platform; no substrate changes silently; every promotion is battery-gated against pre-registered acceptance deltas; and model identity is recorded per turn in the unified turn ledger. Regression batteries are assessed by a different provider from the one that generated the replies under test (Dobson, 2026b). A silent substrate swap is therefore detectable by construction, and substrate effects are separable from scaffold effects in the record, which is the precondition for the ablation predicted as F4 in §4.3. The verdicts in §3.1 are stated at the level of runtime carriers and hold across substrate identity.

#### 2.4.5 Two ledger-verified operational facts

Two operational facts about the deployment are reported here as the new empirical material a reference-implementation paper can properly offer. Both are facts about the deployment rather than task-performance results, and both are obtained by direct query against the production project: the first against the unified turn ledger, which holds one row per assistant turn per surface; the second against the database catalogue. Each is reproducible under the inspection terms in §2.4.6 and interpreted in §4.1.

- Across the 90-day window from 20 May to 17 August 2026 inclusive, the practitioner-facing Companion surface generated 765 substrate turns, all vendor-attributed to OpenAI in the turn ledger’s source field, with zero substrate turns under Anthropic models; three further Companion turns in the window took non-substrate system paths (recall and safety). The Research seat produced its notes under Claude Sonnet 5. The two substrate sets are disjoint by seat design.
- The database role clara_research_readonly holds SELECT-only privileges on exactly five tables in the production project; rolbypassrls is false; write and delete privileges are denied at the RLS layer.

The first fact establishes that the runtime carriers named in §3.1 hold on a surface whose substrate vendor differs from that of the reading layer on which several were exercised during development. The second establishes that the read-only character of the Research seat is enforced by the database’s own privilege system and row-level security layer rather than by convention in application code, which is a materially stronger claim than an assertion about scaffold source.

#### 2.4.6 Scope of disclosure and terms of inspection

Three classes of material are withheld from this paper and scoped to the companion paper described in §4.3: the full type signatures of the LensMove object and the seven constitutional checks; redacted trace records showing individual turns through the runtime; and the self-measurement harness with the pre-registered thresholds on which the eight predictions in §4.3 depend. They are withheld because they are measurement apparatus rather than architecture.

The codebase and the deployed policy set are proprietary artefacts of Astrala Advisory Services Ltd. External reproducibility of the verdicts in §3.1 is supported through the external-audit protocol stated in §4.2.1: on request, and under a non-disclosure agreement, a qualified auditor is granted read access to the commit hash to which the verdicts refer, the file-path map from each LIT principle to its named carrier, the deployed row-level security policy set, and the ledger extracts supporting the two facts in §2.4.5. This form of reproducibility is standard for a systems paper reporting a private deployment of a published specification.

## 3 Specification-conformance evidence

This section reports the paper’s evidence. §3.1 is the specification-conformance report against LIT’s eight design principles, summarised in Table 1. §3.2 applies the same grading discipline to the perceptual control theory commitments the Companion voice borrows, so that the paper’s home theory and its borrowed theory are graded with equal care.

### 3.1 The eight-principle specification-conformance report

This section reports specification conformance rather than empirical evaluation, and the distinction governs what the verdicts can and cannot establish. Each of the eight LIT design principles is stated, assigned a verdict of built at scaffold, partial, or substrate-unreachable, and paired with the named runtime carrier grounding that verdict or the specific gap preventing one. A verdict of built at scaffold asserts that a named, inspectable runtime object implements the principle’s requirement at the scaffold layer; it does not assert that the implementation improves any measured outcome. Verdicts are stated at the level of runtime carriers and therefore hold across substrate identity; where a carrier is evidenced on a particular surface.

The sorting of the eight principles into a parameter-level family and a scaffold-level family is this paper’s analytic contribution rather than a restatement of Dobson (2026a), which states the principles without sorting them by implementability from outside a hosted substrate. Three principles quantify over the model’s own parameters or hidden state and are therefore structurally unreachable from a scaffold; five quantify over the architecture surrounding the model and are implementable at scaffold. The verdicts below follow that sorting.

#### 3.1.1 Associative slack (LIT 5.4.1)

Principle: Preserve associative slack in hidden-state representations so that the model retains capacity for non-obvious associations rather than collapsing onto the most probable continuation. Verdict: substrate-unreachable. The principle quantifies hidden-state covariance during training, and the scaffold has neither write access to the substrate’s parameters nor read access to its internal activations. What the scaffold does instead is preserve associative slack at the level of its own selection behaviour: the lens engine’s dominance guard (§2.2.6) removes an over-represented antagonist cluster from the candidate set, and the silent-lens contract keeps the practitioner’s attention on the question rather than on the framework. Neither is a substitute for the principle as specified, and neither is offered as one; they are scaffold-level analogues at a different layer of the composite system.

#### 3.1.2 Affect-coupled learning (LIT 5.4.2)

Principle: Couple affect to learning, so that uncertainty and surprise signals modulate the model’s updates rather than only its surface behaviour. Verdict: substrate-unreachable. Gradient-coupled affect requires access to the training loop, which a hosted substrate does not expose. The scaffold carries a structural analogue: the register profile (foldReading, PROFILE_ALPHA = 0.2; persisted in clara_register_readings and clara_user_factor_profile) includes an emotional category alongside the cognitive, symbolic, strategic, and ethical categories, and pillar_emphasis carries a session-level reading of what matters to the practitioner now. Both influence which move is selected, so the affect-like signal modulates the system’s behaviour; it does not modulate any parameter update, and the paper does not claim it does.

#### 3.1.3 Contradictions as first-class objects (LIT 5.4.3)

Principle: Represent contradictions as first-class, addressable objects rather than as noise to be smoothed away. Verdict: built at scaffold. The runtime carrier is the typed field LensMove.collapses_antagonist_pair, checked by assertConstitutional under Principle 2, together with the antagonist_cluster string carried by every mechanism-registry card (§2.2.5). A candidate move that would resolve a live antagonist pairing—Sarasvathy against McGrath, Schön against Argyris, the two Cope grains against each other, Vygotsky against Bandura—into a single reconciled position fails the check and does not surface. The contradiction is therefore addressable in the strict sense: it has a name, a typed field, and an assertion that fails closed when it is collapsed. This is the clearest case among the eight of a theoretical commitment carried by an inspectable runtime object.

#### 3.1.4 Reflection as architecture (LIT 5.4.4)

Principle: Institute reflection as architecture rather than as a prompt-level suggestion. Verdict: built at scaffold. Two carriers ground the verdict. The six-stage developmental cycle (§2.2.8) makes Reflection and Repatterning named stages that a practitioner’s work passes through, with a cycle-stage value carried on the candidate move. And clara_aspire_scaffold_traces records an append-only trace row for each stage transition, so a reflection event is a durable record rather than a conversational moment. Reflection here is a state that the architecture requires the work to occupy and writes down when it does, not an instruction that the substrate may honour or ignore. The limitation is that the trace records that a transition occurred rather than what the practitioner understood by it, and the companion paper’s F6 addresses that gap.

#### 3.1.5 Layer-1 compute budget (LIT 5.4.5)

Principle: Expose an explicit compute budget at the model’s first processing layer, readable by a reflective controller. Verdict: partial. The literal requirement is substrate-unreachable, because a hosted API exposes no per-layer compute allocation and no interface for a controller to read or set one. What exists at scaffold is a budget of a different kind: server/lib/engine/profile.ts maintains a per-practitioner engine profile with an exponential smoothing constant PROFILE_ALPHA = 0.2, and the selector’s two-pass structure (server/lib/aspire-v3/lens-selector.ts) uses deterministic selection first and invokes the LLM pass only where the deterministic pass falls silent and the message clears LLM_MIN_MESSAGE_CHARS = 6. A reflective controller therefore does read and act on a resource allocation, at the granularity of which selection path to spend rather than which layer’s compute to allocate. The verdict is partial because the mechanism is real and the layer is wrong.

#### 3.1.6 Post-training substrate plasticity (LIT 5.4.6)

Principle: Provide post-training substrate plasticity, in which the reflective layer feeds back into durable parameter change over long timescales. Verdict: substrate-unreachable. Parameter change requires write access to weights. The scaffold’s durable change is written to its own state rather than to the substrate’s: the register profile updates as sessions accumulate; the Bayesian thinker-weights posterior (clara_aspire_thinker_weights) updates from typed feedback events—a completed pillar feedback reinforces the thinkers surfaced in that cycle—and the engine’s selection ordering is posterior-weighted when weights are supplied, with the write path live at the audit basis, the posterior table unpopulated pending the first feedback events, and the turn path not yet supplying weights, so all practitioners remain on the uniform prior and selection is unchanged; and the memory layers described in §2.4.3 consolidate at their own cadences. A practitioner returning after a month meets a system whose record of them has changed; the substrate behind that system has not. The distinction matters because conflating the two would allow any system with a database to claim substrate plasticity.

#### 3.1.7 Multi-timescale coupling (LIT 5.4.7)

Principle: Couple learning across inference, episode, and consolidation timescales. Verdict: built at scaffold, implicit. The seven memory layers (§2.4.3) operate at genuinely different cadences: the turn-level layers within a single exchange, the session and thread layers within an episode, and the Portrait and team-pattern layers across consolidation intervals. The carriers are the seven layers themselves, together with the access-plane separation enforced at the schema level. The verdict is qualified as implicit because the separation of timescales is a property of which table writes when, rather than an explicit typed field that a check can interrogate; no assertion reads a timescale value, because none exists. Making the timescale explicit is designated as a v-next item in §4.2.6.

#### 3.1.8 Falsifiability and empirical agenda (LIT 5.4.8)

Principle: State a falsifiable empirical agenda: theory-level predictions that would count against the architecture if unmet. Verdict: built at scaffold, forward-looking. The carrier is the pre-registered prediction set F1 to F8 stated in §4.3, each with a named measurement instrument, a pre-registered threshold, and an explicit falsification condition. The measurement harness itself is scoped to the companion paper, and this paper therefore reports the agenda rather than its results. The verdict records that the agenda exists in falsifiable form and is committed to in advance of the data, which is what the principle requires of the architecture; whether the predictions survive contact with the data is the companion paper’s question and not this one’s.

Table 1 records, for each of the eight LIT design principles, the verdict, the named runtime carrier or the gap preventing one, and the surface on which the carrier is evidenced. The verdict key is as follows. “Built at scaffold” is used where a named, inspectable runtime object—a typed field, a function, or a database table—implements the principle’s requirement at the scaffold layer, and where that object can be located in the codebase or the production schema at the audit basis stated in §2.4. “Partial” is used where a mechanism of the right kind exists at scaffold but operates at a different layer of the composite system from the one the principle specifies, so that the requirement is met in form but missed in location. “Substrate-unreachable” is used where the principle quantifies over the substrate’s parameters or internal activations, to which the scaffold holds neither write nor read access, and where no scaffold-level construction could satisfy the requirement as stated without a change in the substrate interface.

The final column records which of the four surfaces disclosed in §2.4.4 evidences each verdict, since the reference implementation reported in this paper is the platform as a whole rather than any single surface. Where a verdict is substrate-unreachable, the column records the surface on which the scaffold-side analogue runs and states that no surface evidences the clause itself, so that the two claims are not conflated.

### 3.2 PCT primitives as prompt-level companion voice discipline

This section reports how perceptual control theory (PCT) and the method of levels (MoL) shape the Companion’s conversational voice, using the same built at scaffold, partial, and substrate-unreachable grading discipline that §3.1 applies to the eight LIT principles.

#### 3.2.1 Grading stance and scope

This section applies the same verdict discipline to perceptual control theory. The grading is deliberately conservative: PCT primitives shape the Companion’s conversational register through the system prompt, and none is enforced by a runtime assertion analogous to assertConstitutional. Reporting them as implemented mechanisms would overstate the architecture, so each is reported as prompt-level voice discipline, and the gap to a runtime carrier is identified.

#### 3.2.2 What is borrowed, and at which layer it operates

Perceptual control theory holds that behaviour is the control of perception: an organism acts to keep a perceived variable near an internal reference value, and adaptive change occurs through the reorganisation of the control hierarchy rather than through the accumulation of responses (Powers, 1973; Marken, 2014). The method of levels is the associated clinical technique, in which attention is directed to the level above the one currently in conflict so that the conflict can reorganise (Carey, 2006). Both inform the Companion’s register: its willingness to hold a live conflict in view, its preference for a question that opens a level over an answer that closes one, and its refusal to interpret mid-reorganisation disturbance as decline. This is a real and documented design influence, operating through instructions to the substrate rather than through a checked invariant. Four of the seven commitments constituting the platform’s PCT-Accuracy Criterion—commitments 3, 4, 5, and 7—received scholarly review from advisors within the perceptual control theory community in July 2026; the contribution was the review of commitments already built at that date, not architectural co-design.

#### 3.2.3 The controlled variable: no runtime representation

The controlled variable, the perception an organism acts to keep near reference, has no runtime representation. The Companion is instructed to attend to what a practitioner appears to be holding steady, and its questions are shaped accordingly, but no field on any object names a controlled variable and no assertion checks that a candidate move respects one. The gap to a runtime carrier is specific: a typed controlled_variable field on the candidate move, populated by inference from session history and checked before composition, would shift this from voice discipline to mechanism. It is named as a v-next item in §4.2.6.

#### 3.2.4 The portrait’s abstention from reference-value storage

The reference value is likewise absent as a runtime object, and the Portrait’s design makes this deliberate rather than accidental. The register profile stores a dispositional signature of how a practitioner moves under decision pressure, not the specific values they are trying to hold steady at a given time, precisely because freezing shifting reference values into a static record would misrepresent them (§2.2.2). The consequence is that the architecture holds no reference-value construct at all. The accurate reading is that PCT’s caution was taken seriously at the level of what not to store, and the corresponding positive construct was never built.

#### 3.2.5 Two different taxonomies, not a mapping

PCT specifies a hierarchy of perceptual control at 11 levels, from intensity through sensation, configuration, transition, event, relationship, category, sequence, program, principle, to system concept (Powers, 1973). The Portrait’s five categories—cognitive, emotional, symbolic, strategic, ethical—are not a mapping onto those levels, and the paper does not claim they are. They represent a separate taxonomy serving a different purpose: describing dispositional movement for lens selection rather than modelling levels of perceptual control. A reader familiar with PCT should not interpret the five categories as a compressed 11.

#### 3.2.6 Reorganisation and repatterning: thematic, not mechanical

Reorganisation, the mechanism by which a control hierarchy changes when persistent error cannot be reduced within the existing structure, is the PCT construct closest to the six-stage cycle’s Repatterning stage, and the correspondence is thematic rather than mechanical. Repatterning is a named stage with a trace row; reorganisation is a hypothesised process in a control hierarchy with no counterpart in the scaffold’s state. The going-up-a-level move shapes the Companion’s questions and is not implemented as a runtime operation on any hierarchy. Both would require a control-hierarchy representation that the architecture does not have before they could be reported as mechanism.

#### 3.2.7 MYLO citation discipline

MYLO’s published trial evidence—Cohen’s d in the region of −0.4 to −1.1 across replications on problem distress, anxiety, and psychiatric impairment (Gaffney et al., 2014; Wrightson-Hester et al., 2023, 2025)—supports the mechanism family the Companion voice draws on: curious, attention-orienting, MoL-style questioning. It does not support the specific implementation reported in this paper. The Companion does not use MYLO’s curated question bank, has not been the subject of a trial, and does not claim clinical equivalence with MYLO’s evidence base.

## 4 Discussion

This section discusses what the theory constrained about the build and what the build revealed about the theory (§4.1), states the limitations of the work plainly together with the external audit protocol that would relax the principal one (§4.2), and names the companion paper in which the empirical evaluation of the deployed system is specified, with its eight falsifiable predictions enumerated (§4.3).

### 4.1 Interpreting the reference implementation

Three aspects are discussed here in turn: what LIT’s specification constrained about the engineering, what the engineering taught about the specification, and what the two ledger-verified facts reported in §2.4.5 show about the locus of the architecture.

#### 4.1.1 What the theory constrained

Two constraints imposed by the theory deserve mention because in both cases the specification ruled out an implementation that would otherwise have been the obvious choice. LIT’s contradiction principle ruled out the reconciling assistant: the natural instinct when two frameworks pull in opposite directions is to synthesise a position the practitioner can act on, and Principle 2 forbids it at composition time. PCT’s caution about reference values ruled out the goal-tracking Portrait: the natural instinct is to record what a practitioner is trying to achieve and measure progress against it, and instead, the dispositional signature was built. Neither constraint was discovered through building; both were imposed by the specification before the relevant code existed.

#### 4.1.2 What the build taught about the theory

The most consequential revelation from the build is that a fail-closed assertion at prompt-composition time is a different kind of object from a system-prompt instruction, and that the difference is architectural rather than stylistic. A system prompt asks the substrate to behave; an assertion removes a candidate move from the pipeline before the substrate is asked anything. The two are frequently discussed as points on a single spectrum of strictness, but they are not: one is a request whose compliance rate is an empirical question, while the other is a gate whose behaviour is a property of the scaffold’s control flow.

This is why the paper reports six of the seven checks as carrying a formal guarantee relative to their declared annotations, and one as carrying an empirical one (§2.3.2). A typed assertion over a structured field classifies nothing and therefore has no error rate of its own; a pattern-matched detector over free text classifies and therefore does. Collapsing the two categories would allow the weaker guarantee to borrow the stronger one’s credibility, which is precisely the move a reader of an architecture paper should be able to check.

The corollary for practitioners building over hosted substrates is that the enforcement point is a design variable, and the earliest available one is composition. Everything the scaffold can decide before the substrate call is decidable with scaffold-level guarantees; everything deferred until after the call is subject to the substrate’s behaviour. The two ledger-verified facts in §2.4.5 bear on this: the runtime carriers hold on a surface whose substrate vendor differs from that of the reading layer, which is what one would expect of enforcement located in the scaffold rather than in the model.

#### 4.1.3 Change control as a load-bearing guarantee

A runtime carrier is only as strong as the guarantee that its schema will not drift, and the two-phase migration apply-guard described in §2.4.1 is therefore load-bearing for the verdicts in §3.1. A verdict of built at scaffold cites a table or a typed field at a stated commit; without a mechanism that fails the build when a migration is unledgered, that citation degrades quietly over time. Specification-conformance claims about a live system therefore require change-control evidence alongside the architectural description.

#### 4.1.4 Locating enforcement in the data layer

The read-only Research seat, reported in §2.4.5 as the second ledger-verified fact, was built to answer a question about the architecture rather than about the product: whether a seat could read the shared memory layers for research purposes without any write path, enforced by the database’s own privilege system rather than by convention in application code. The answer is that it can, and the enforcement is stronger than a scaffold-level claim because it does not depend on the correctness of the application. Some architectural commitments are therefore better located in the data layer than in the code layer, and a paper reporting runtime carriers should say which layer each commitment lives in.

#### 4.1.5 A negative result: three principles stayed unreachable

Three of the eight principles are substrate-unreachable, and no amount of scaffold ingenuity changed that during the build. Attempts to construct scaffold-level analogues for associative slack, affect-coupled learning, and post-training plasticity produced mechanisms that were real, useful, and at the wrong layer, which is why §3.1 reports them as analogues rather than as implementations. A specification written for a system with parameter access cannot be fully satisfied by a system without it, and the useful response is to name the boundary rather than to blur it.

#### 4.1.6 What a reference-implementation paper can claim

This paper’s evidence is a mapping from published specification clauses to named, inspectable runtime carriers, plus two facts about the deployment. It does not show that the architecture produces better outcomes for practitioners, that the constitutional runtime reduces prescriptive language in deployed conversations, or that the developmental cycle changes how practitioners work. Each is a measurement question with a stated instrument and a pre-registered threshold in the companion paper (§4.3). The claim here is narrower and, within its scope, checkable: that these commitments have carriers, that the carriers can be located, and that three of the eight principles cannot be carried at all from where this implementation sits.

The genre distinction is worth defending because reviewers reasonably ask why the two are not one paper. Specification conformance and empirical evaluation answer different questions and fail in different ways. A conformance report fails when a cited carrier does not exist, does not do what the specification requires, or has drifted from the commit at which it was cited; those failures are found by inspection. An evaluation fails when an effect is absent, smaller than pre-registered, or confounded; those failures are found by measurement. Combining them allows the weaker evidence to borrow from the stronger presentation.

### 4.2 Limitations and future work

Nine limitations are stated, with the external-audit protocol first because it conditions the others. Each names what a sceptical reader cannot currently check and what would be required to make it checkable.

#### 4.2.1 External-audit protocol and the self-authorship limitation

The verdicts in §3.1 cite runtime carriers in a proprietary codebase, and a reader cannot open the files. The protocol offered is the one stated in §2.4.6: on request, and under a non-disclosure agreement covering commercially sensitive material, a qualified auditor is granted read access to the commit hash to which the verdicts refer, the file-path map from each LIT principle to its named carrier, the deployed row-level security policy set, and the ledger extracts supporting the two facts in §2.4.5. The audit basis is fixed at repository commit d9259cb807c6d965ed2d7f5be264262ef9d48883 and the production database as of 19 August 2026, so an auditor checks the same artefacts the verdicts were written against rather than a later state of the system. This is weaker than an open repository and stronger than an unverifiable description.

The specification against which conformance is reported was authored by the present author, and the two are not independent. The mitigation adopted throughout is procedural rather than substantive: the specification is published separately and in advance (Dobson, 2026a), its clauses are restated rather than reinterpreted in §3.1, and each verdict cites a carrier that a third party could inspect. What remains unmitigated is that a specification written by the same author who built the implementation may have been shaped, at the margin, by what turned out to be buildable.

#### 4.2.2 Falsifiability protocol authored elsewhere

The ledgers described in §3.1.8 capture data sufficient to test several of the predictions enumerated in §4.3 using a matched-cohort design and a pre-specified primary outcome. The predictions and their instruments are specified in the companion paper; the matched-cohort clinical protocol itself—cohort matching criteria, primary outcome, stop rule—remains future work for both papers and is treated as a research design deserving of its own care rather than a routine engineering ticket.

#### 4.2.3 Selector budget signal captured but not read

latency_ms and model_used appear in every selector decision row (§3.1.5). No reflective controller currently reads them and modulates selection behaviour accordingly. This is held pending a concrete learner-facing reason to throttle selection based on compute cost, rather than being built speculatively ahead of that need.

#### 4.2.4 Verdicts are claims about a stated state

The verdicts are stated at a single commit and a single database state. A live platform changes, and a carrier cited today may be refactored tomorrow. The mitigation is the audit basis stated above and the migration apply-guard described in §2.4.1, which fails the build when a schema change is unledgered; neither prevents a future divergence between the paper and the system, and a reader should treat the verdicts as claims about a stated state rather than about the platform in perpetuity. The verdicts are also stated at the deployment’s current scale: they concern the existence and location of runtime carriers, which are scale-independent properties, and make no claim about the system’s behaviour, latency, or enforcement characteristics under load.

#### 4.2.5 Annotation fidelity is unmeasured

Four of the six typed assertions read declared annotations—the authority flag, the developmental-fit delta, the static-hierarchy flag, and the elegance-justification flag—that the current move composer does not yet populate; until estimators exist, those checks pass vacuously, and the single documented landing site for the estimators is the move-construction helper on the compose path. The pattern-matched detector and the two populated typed checks carry the enforcement burden at the audit basis. The Bayesian thinker-weights posterior sits in the same category: its feedback write path and its weighted read are live at the audit basis, and the posterior table is unpopulated pending the first feedback events, so selection remains at the uniform prior. This is stated so that the formal-guarantee claim in §2.3.2 is understood at its true strength.

#### 4.2.6 PCT runtime enforcement absent; named v-next carriers

The Companion voice’s PCT-shaped conflict-orientation is prompt-level discipline, checked by human review of transcripts, not a runtime-checked representation (§3.2). Three v-next carriers are named so that the gaps are specific rather than gestural: a typed controlled_variable field on the candidate move, populated by inference from session history and checked before composition (§3.2.3); a runtime conflict detector symmetric in shape to the PrescriptiveLanguageDetector, which would first require specifying how a live conflict between two references is represented or inferred from an exchange; and an explicit timescale field on the memory layers, which would convert the implicit coupling reported in §3.1.7 into a typed one.

#### 4.2.7 Inter-principle conflict resolution is priority-ordered, not adjudicated

As stated in §2.3.5, the seven constitutional principles are resolved by strict priority order—Principle 5 is dominant, with other conflicts defaulting to fail-closed rejection—rather than by a mechanism that adjudicates between competing principles based on their merits in a given case. This is a known limitation shared with Constitutional-AI-style systems generally, and the paper does not claim to have solved it.

#### 4.2.8 Deployment observations held for a follow-up paper

This paper reports architecture only. Clara Nexus is, by the company’s own internal strategic description, a live and revenue-generating platform, and usage patterns, practitioner outcomes, and retention data exist. Reporting that data alongside the architecture would conflate two different epistemic exercises—describing what was built and evaluating what happened when practitioners used it—and this paper defers the second to a dedicated follow-up.

#### 4.2.9 Language and locale

The Companion voice is English-native. Greek-native pillar prompts, corresponding to the entrepreneurial pillars described in §2.2.4, have been drafted but await sign-off from a native philosophical reader before being treated as production-ready; the paper does not report on multilingual performance because that work has not yet reached a state suitable for reporting.

### 4.3 The companion paper and its falsifiable predictions

The empirical evaluation of the deployed system belongs to a companion paper in preparation for submission to Frontiers in Artificial Intelligence, provisionally titled Measuring a Scaffold from Inside: An Operator-Authored Self-Measurement Harness for a Constitutional Runtime over a Hosted Language Model (Dobson, in press). It is named here, with its predictions enumerated, so that the empirical follow-through this paper defers is directed towards a stated target with explicit commitments rather than to an unspecified future. The runtime carriers named in §3.1 are the objects of that measurement, and the eight predictions below are the empirical commitments this paper makes on their behalf. Each carries a pre-registered falsification condition.

F1—constitutional-clause referencing incidence in the practitioner-facing surface: the rate at which turns reference a constitutional clause. Falsified if the observed incidence does not exceed the pre-registered floor.

F2—substrate-call latency budget adherence: the proportion of turns whose substrate call completes within the pre-registered latency budget. Falsified if adherence falls below the stated threshold.

F3—PrescriptiveLanguageDetector recall, precision, and F1 measured on a pre-registered hand-labelled corpus. Falsified if any of the three falls below its pre-registered floor. This is the prediction that supplies the false-positive and false-negative characteristics of the one pattern-matched check among the seven, as noted in §2.3, and the prediction under which annotation fidelity for the typed checks is measured (§4.2.5). An observe-only production detector began logging severity-warn rows against live surfaced replies on 19 August 2026, without altering any reply; those rows constitute the hand-labelling corpus for F3.

F4—The constitutional runtime’s effect on prescriptive-language incidence, measured before and after the ablation of the runtime. Falsified if incidence does not differ in the predicted direction by the pre-registered margin. F4 requires an ablation switch for the constitutional runtime; the switch is measurement apparatus and is scoped to the companion paper.

F5—Portrait-referencing incidence and its consistency across sessions. Falsified if referencing incidence or cross-session consistency falls below the pre-registered floor.

F6—Decision continuity across sessions when the Portrait is populated, compared with a baseline in which it is not. Falsified if continuity does not differ in the predicted direction.

F7—Reflection-cycle completion incidence and its effect on the constitutional-conformance rate of the next move. Falsified if completion incidence falls below its pre-registered floor, or if the conformance effect is absent.

F8—Reflection-driven revision of Portrait entries as a marker of within-session learning. Falsified if revision events do not occur at the pre-registered rate, or do not follow reflection events.

Two scope statements complete the division. First, the boundary is drawn where architecture ends and measurement begins: type signatures, redacted trace records, and the self-measurement harness itself are scoped to the companion paper (§2.4.6), because presenting measurement apparatus in an architecture paper invites the reader to treat conformance verdicts as performance results. Second, both papers are authored by the present author, and the operator-authored character of the measurement harness is named as a limitation in the companion paper on the same grounds stated in §4.2.1; the external-audit protocol described there applies to both.

## 5 Conclusion

The engineering claim of this paper is narrow and, within its scope, checkable. A published specification for a class of cognitive architecture can be implemented over a hosted frontier language model substrate to the extent that its clauses quantify over the scaffold rather than over the substrate’s parameters, and the boundary between the two is not a matter of effort but of interface. Four of the eight LIT design principles have named runtime carriers at the scaffold layer; one is partial, met in form and missed in location; three are substrate-unreachable and are reported as such rather than approximated. The constitutional runtime is the core contribution: seven fail-closed checks at prompt-composition time, six typed and one pattern-matched, enforced before any substrate call and therefore auditable independently of whichever model sits behind the API.

What follows from this is a reporting standard rather than a result. A system that describes itself in the vocabulary of cognitive architecture should be able to name, for each commitment it claims, the runtime object that carries it, the layer at which that object resides, and the commitments it cannot support from that position. Specification conformance reported in this manner is checkable by inspection and separable from the question of whether the architecture functions, which is a measurement question addressed in the companion paper mentioned in §4.3.

## Data Availability

The original contributions presented in the study are included in the article/supplementary material. External reproducibility of the specification-conformance verdicts in §3.1 is supported through the external-audit protocol described in §2.4.6 and §4.2.1: on request, and under a non-disclosure agreement, a qualified auditor is granted read access to the audit basis (repository commit d9259cb807c6d965ed2d7f5be264262ef9d48883 and the production database as of 19 August 2026), the file-path map from each LIT design principle to its named runtime carrier, the deployed row-level-security policy set, and the ledger extracts supporting the two operational facts in §2.4.5. Further inquiries can be directed to the corresponding author.
